# Hepatic Anæmia

**Published:** 1880-06

**Authors:** 


### HEPATIC ANÆMIA.

There is an hepatic anæmia, that is, an anæmia produced by hepatic disorder. This form of anæmia is remarkably common, in fact the commonest form; and all cases of anæmia without any apparent cause are hepatic. The alkalies, especially potash, have a beneficial action on the liver, which action tends to restore the blood to its normal character. The alkalies ought to take the place of iron in the treatment of anæmia. Potash has a much greater affinity for oxygen than soda out of the system, and probably it is the same inside the system. In using the alkalies we must not limit ourselves to symptoms localized in the digestive track, such as a furred tongue, loss of appetite, acid eructations, flatulence, heartburn, etc., for there are conditions where none of these symptoms are present, and yet where the alkalies are beneficial and curative. Perhaps the best known indication that is not local, consists in the abnormal urinary sediments, whose formation is prevented by alkalies. It is the same often with gouty pains, and with many cases of eczema and other skin eruptions. The morbid condition is caused by a disorder of the liver, which is not directly connected with digestion so much as with the blood. We may give the alkalies to improve the tone of the digestive system, increasing the appetite, aiding the liver to work, promoting the flow of bile, and clearing the blood and urine from lithates, or sediments and impurities. If the alkalies can effect this, then assuredly they are tonic. The pigment of the urine is derived from the biliary pigment; and the sediments, from the digestive tract, or, as Murchison more correctly limits it, from the liver. If the alkalies clear the urine from these pigments and deposits, it must be by virtue of a salutary action on the liver. Certainly the most important action of potash is on the liver, and especially on that function of disintegration on which excretion depends, and the elimination of bile, consequently the good effects are seen, not locally only, but universally. In what manner it acts on this organ may be questionable; perhaps the oxidation theory may be correct. In anæmia the best results are found from potash from the beginning to the end of the disease, and by it the author hopes to cure most of the cases he treats, unless due to tubercle, or secondary to some incurable lesion. The alkalies produce no depressing action; but when given continuously for long periods the patient or the patient's stomach sickens at them, the same as it might do at any other monotony. Dr. Nicholson is in the habit of prescribing the bicarbonate of potash in twelve to twenty-grain doses, four times a day, continuously for months, combining with it the spirit of chloroform, which enables it to be better borne. He has failed to trace any connection between pyrosis and the continuous administration of potash, and regards the idea as traditional and not supported by unbiased observation.—*New York Medical Journal.*

